# Pre-Pregnancy Obesity, Excessive Gestational Weight Gain, and the Risk of Pregnancy-Induced Hypertension and Gestational Diabetes Mellitus

**DOI:** 10.3390/jcm9061980

**Published:** 2020-06-24

**Authors:** Małgorzata Lewandowska, Barbara Więckowska, Stefan Sajdak

**Affiliations:** 1 Medical Faculty, Lazarski University, 02-662 Warsaw, Poland; 2 Division of Gynecological Surgery, University Hospital, 33 Polna Str., Poznan University of Medical Sciences, 60-535 Poznan, Poland; ssajdak@ump.edu.pl; 3 Department of Computer Science and Statistics, Poznan University of Medical Sciences, 60-806 Poznan, Poland; barbara.wieckowska@ump.edu.pl

**Keywords:** obesity, weight gain, pregnancy, hypertension, preeclampsia, diabetes, overweight, risk

## Abstract

Excessive pre-pregnancy weight is a known risk factor of pregnancy complications. The purpose of this analysis was to assess the relationship between several categories of maternal weight and the risk of developing hypertension and diabetes in pregnancy, and the relationship of these complications with the results of the newborn. It was carried out in a common cohort of pregnant women and taking into account the influence of disturbing factors. Our analysis was conducted in a prospective cohort of 912 Polish pregnant women, recruited during 2015–2016. We evaluated the women who subsequently developed diabetes with dietary modification (GDM-1) (*n* = 125) and with insulin therapy (GDM-2) (*n* = 21), as well as the women who developed gestational hypertension (GH) (*n* = 113) and preeclampsia (PE) (*n* = 24), compared to the healthy controls. Odds ratios of the complications (and confidence intervals (95%)) were calculated in the multivariate logistic regression. In the cohort, 10.8% of the women had pre-pregnancy obesity (body mass index (BMI) ≥ 30 kg/m^2^), and 36.8% had gestational weight gain (GWG) above the range of the Institute of Medicine recommendation. After correction for excessive GWG and other confounders, pre-pregnancy obesity (vs. normal BMI) was associated with a higher odds ratio of GH (AOR = 4.94; *p* < 0.001), PE (AOR = 8.61; *p* < 0.001), GDM-1 (AOR = 2.99; *p* < 0.001), and GDM-2 (AOR = 11.88; *p* <0.001). The threshold risk of development of GDM-2 occurred at lower BMI values (26.9 kg/m^2^), compared to GDM-1 (29.1 kg/m^2^). The threshold point for GH was 24.3 kg/m^2^, and for PE 23.1 kg/m^2^. For GWG above the range (vs. GWG in the range), the adjusted odds ratios of GH, PE, GDM-1, and GDM-2 were AOR = 1.71 (*p* = 0.045), AOR = 1.14 (*p* = 0.803), AOR = 0.74 (*p* = 0.245), and AOR = 0.76 (*p* = 0.672), respectively. The effect of maternal edema on all the results was negligible. In our cohort, hypertension and diabetes were associated with incorrect birth weight and gestational age at delivery. Conclusions: This study highlights the importance and influence of excessive pre-pregnancy maternal weight on the risk of pregnancy complications such as diabetes and hypertension which can impact fetal outcomes.

## 1. Introduction

According to global estimates, the incidence of obesity has doubled in the last four decades, and over 30% of women are obese [1]. Because adipose tissue is an active metabolic organ, it is not surprising that obesity increases the risk of metabolic disorders, including cardio-vascular diseases (in the general population) [1,2]. Many studies have shown that overweight and obesity before pregnancy increase the risk of main pregnancy complications such as hypertension and diabetes mellitus in pregnancy [3,4,5,6]. Both complications are associated with a higher incidence of maternal and neonatal complications (morbidity and mortality), including a higher incidence of offspring with metabolic diseases in adult life [4,7,8,9,10].

Many studies have shown that pre-pregnancy obesity and overweight are independent pregnancy-induced hypertension (PIH) and gestational diabetes mellitus (GDM) risk factors [6]. In most studies, maternal obesity was the strongest risk factor, but in several studies overweight was more likely than obesity to increase the risk of these complications [11,12,13,14,15]. Relationships between gestational weight gain (GWG) above the recommendation and a higher risk of pregnancy-induced hypertension (PIH) as well as a lower risk of gestational diabetes (GDM) were found [6,16,17]. However, the relationships between excessive GWG and GDM are divergent [18]. The available studies differ regarding the size of the groups studied, the degree of correction, and, in some cases, the definitions of the body mass index (BMI) categories. Some studies evaluated all cases of hypertension in pregnancy including pre-existing hypertension, while other studies only assessed preeclampsia. Not all obesity studies have considered the effect of gestational weight gain. An important confounding factor overlooked in the studies is maternal edema; it is not certain to what extent weight gain is the result of increased body fat, and to what extent the result of fluid retention.

The aim of our study was to check the relationship between various maternal weight categories and the risk of developing pregnancy complications such as hypertension or diabetes, which in turn could affect the results of the newborn. We evaluated the main forms of these diseases separately. Our goal was to take into account important disturbing factors including maternal edema. We excluded women with pre-existing hypertension or diabetes.

## 2. Materials and Methods

The entire research process carried out to implement this analysis was in line with the Helsinki Declaration. The women recruited for the study were informed about the voluntary character of their participation. All recruited participants read and signed the Conscious Consent Form. The study was approved by the Bioethics Committee of the Medical University of Poznan (Poland) (769/15).

### 2.1. Study Population and Method

We examined a cohort of pregnant women who were enrolled at the Obstetric-gynecological and Neonatological Hospital (in Poznan, Poland). This is the university hospital with the highest degree of references for obstetric cases, in which the number of deliveries per year is about 7000. The recruitment process was carried out during 2015–2016.

Polish white women from the Wielkopolska region were recruited (white but not Polish women were not included because their number is small in our country). Other inclusion conditions involved single pregnancy, gestational age between 10th and 14th week, no fetal aneuploidy, gestational age of delivery ≥ 25th week, and delivery of a phenotypically normal child. The inclusion criterion was also the mother′s age during conception of 18–45 years.

We excluded women with chronic diseases, primarily diabetes mellitus, and/or arterial hypertension. Kidney disease and/or liver disease were excluded as well. The same regarded thromboembolism and diseases related to immune disorders. Excessive body mass index on the other hand was not an exclusion criterion.

Information related to the characteristics of the participants was collected during recruitment. For this purpose, we used a questionnaire created specifically for the purposes of our study. The women completed these questionnaires in the presence of a medical worker (midwife), but did it on their own. They were asked about their age, height, and weight before pregnancy. The following questions concerned the course of the current pregnancy as well as the number and history of previous births. We collected information about the multivitamin preparations and other drugs they took. We asked them about addictions such as smoking, drinking alcohol, and other drugs (alcohol and other drugs were not used during pregnancy). We also collected the information about other previous illnesses and operations. An important part of the questionnaire was questions about socio-economic indicators and demographic data.

The data we collected after the end of pregnancy were taken from medical records. Information on pregnancy outcomes included fetal sex, birth weight, gestational age at birth, intrauterine growth restriction, and APGAR (an assessment of appearance, pulse, grimace, activity, and respiratory) results. The information on pregnancy complications mainly included the development of gestational diabetes mellitus (GDM) and pregnancy-induced hypertension (PIH). The information on mother′s edema was also recorded.

The PIH covered the development of de novo hypertension after 20th gestational week and its resolution within 12 weeks after delivery. Hypertension was defined as a pressure value ≥ 140/90 mmHg (on two occasions, at least 4 h apart). The pressure was measured in a sitting position, and an oscillometric device was used to measure the pressure. Isolated gestational hypertension (GH) (a milder form of hypertension in pregnancy) was diagnosed if no organ disorder was found. A severe form of the PIH, or preeclampsia (PE), was defined as hypertension accompanied by organ disorders involving the kidneys and/or liver and/or brain, as well as pulmonary edema. Proteinuria was not a mandatory criterion [7]. In all women in our cohort, the diagnosis included only proteinuria ≥ 0.3 g/L. Our definition of PE did not cover the recognition of intrauterine growth restriction. We recorded the values of mothers’ arterial pressure measured in the postpartum ward. The pre-pregnancy pressure was self-reported.

The definition of GDM was in accordance with national and international guidelines (the International Association of Diabetes and Pregnancy Study Groups). This definition was based on the “a 2-h 75 g oral glucose tolerance test (OGTT)”. This is a test performed on pregnant women after an overnight fast. This test is performed between the 24th and 28th gestational week [19]. Two forms of gestational diabetes were diagnosed: diabetes without insulin (GDM-1) and diabetes requiring insulin (GDM-2).

The participants of the study were recruited in hospital laboratory. Brief information about the study and recruitment criteria was posted in the laboratory. In total, 1300 women volunteered for the study over a 12-month period during recruitment during 2015–2016, of whom 388 participants were excluded after the end of pregnancy (when no data on pregnancy results were available, fetal defects were diagnosed, hypertension was diagnosed before the 20th week, and/or diabetes was diagnosed before the 18th week). Finally, the studied cohort consisted of 912 participants.

In the current study, two large analyzes were conducted. In the first analysis, the study group included the women who subsequently developed pregnancy-induced hypertension (PIH) (*n* = 137) (113 gestational hypertension (GH) cases and 24 preeclampsia (PE) cases). Controls for the cases (*n* = 775) were the participants who remained normotensive. In the second analysis, the study group included the women who subsequently developed gestational diabetes mellitus (GDM) (*n* = 146) (125 cases of diabetes with dietary modification and 21 cases of diabetes with additional insulin therapy). Controls for the cases (*n* = 766) were the participants without diabetes.

### 2.2. Studied Variables

The participants reported their pre-pregnancy weight and height on their own. However, the study included growth values recorded in the medical history (based on measurements after admission to hospital). Body mass index (BMI) was estimated for each participant. The formula for calculating BMI was typical: weight (in kilograms) divided by height (in meters) squared. BMI definitions were in line with the recommendations of the World Health Organization (WHO). The BMI definition within the standard range included values of 18.50–24.99 kg/m^2^. Underweight, overweight, and obesity were defined as BMI < 18.50 kg/m^2^, BMI within 25.00–29.99 kg/m^2^, and BMI ≥30 kg/m^2^, respectively.

Maternal weight before delivery (values available in the hospital records) was used to calculate weight gain (GWG). To obtain the GWG value, the weight before pregnancy was subtracted from the weight before delivery. The optimal GWG ranges were different for each BMI category: it was 12.5–18 kg for underweight women before pregnancy; 11.5–16 kg for the women with BMI in the normal range; 7–11.5 kg for women with overweight before pregnancy; and 5–9 kg for obesity before pregnancy. The ranges were defined in line with the recommendations of the (US) Institute of Medicine-IOM from 2009.

We also included information on swelling in women (most often edema of the lower limbs), which was noted in medical reports.

### 2.3. Statistical Analyses

We performed statistical analyses using the Statistica 13 software (TIBCO Software Inc, 2017). The Shapiro–Wilk test was used to assess the normality of the data distribution. We used the Mann–Whitney U test (because normality assumption was not met) for comparisons of continuous variables (means, standard deviation (SD), ranges, and medians are given as description). For categorical ordered categories, the Cochran–Armitage test for trend was applied. For binomial categories, the Pearson chi-square test (or Fisher exact test when Cochran assumption was not met) was applied. *p*-Value< 0.05 was assumed as significant.

The diseases risk for pre-pregnancy BMI and gestational weight gain (GWG) categories was assessed in the logistic regression. The Wald test was used to estimate *p*-value (*p* < 0.05 was assumed to be significant). The whole cohort and the subgroups (the subgroup of GWG in the range of the IOM recommendations, and the subgroup of the normal BMI) were assessed.

Odds ratios of the diseases (and confidence intervals (95%)) were determined for each pre-pregnancy BMI category with respect to the normal BMI (reference category; OR = 1) as well as for GWG above (or below) the IOM recommendation with respect to the GWG in the range (OR = 1). Crude odds ratios (OR) were established in the univariate logistic regression. Adjusted odds ratios (AOR) were obtained after correcting for confounders in several models. The investigated variable was always excluded from confounding variables.

The risk of hypertension in pregnancy was adjusted for hypertension in previous pregnancies, smoking, infertility treatment, age, being primiparous, and for gestational weight gain (GWG) above the range (when BMI was investigated) or for BMI before pregnancy (when GWG was investigated). The risk of gestational diabetes mellitus was adjusted for diabetes in previous pregnancies, maternal age, being primiparous, and for gestational weight gain (GWG) above the range (when BMI was investigated) or for BMI before (when GWG was investigated). The risk results after adjustment for edema are presented in the supplement.

Graphs of risk profiles (in the Lowess method) for pre-pregnancy BMI are presented.

## 3. Results

General maternal characteristics are presented in Table S1. In the whole cohort, the mean mother′s age of participants was 33.7 years (range 18–45 years) and the mean pre-pregnancy body mass index (BMI) was 23.8 kg/m^2^ (range 14.6–42.9). The median of gestational weight gain (GWG) was 13.8 (25–75% ranges 10–17). Overall, 65.0% of the women had normal pre-pregnancy BMI (18.50–24.9 kg/m^2^) and 10.8% were obese (BMI ≥ 30 kg/m^2^). In the cohort, 37.1% of the women had GWG in the range of the IOM recommendations (regardless of the pre-pregnancy BMI category) and 36.8% had GWG above the range. In medical records, edema was reported in 43 (4.7%) women.

Table S1 shows that the women in the case groups (GH, PE, GDM-1, and GDM-2) were older, and more often they were primiparous as well as they more often reporting hypertension or diabetes in previous pregnancies.

Table 1 shows that newborn outcomes differed between women in case groups compared to their controls. Diabetes cases were associated with a higher incidence of birth weight > 90th percentile. Hypertension cases were strongly associated with a higher incidence of birth weight < 10th percentile. GH, PE, and GDM-2 cases were associated with a lower gestational age of delivery.

Relationships between pre-pregnancy BMI and the newborn outcomes are presented in Table S2. Excessive BMI (≥ 25 kg/m^2^) was associated with a higher risk of birth weight > 90th percentile (AOR = 1.69; *p* = 0.031) and > 4000 g (AOR = 1.83; *p* = 0.016), as well as with a higher risk of preterm birth < 34th week (AOR = 3.22; *p* = 0.028). The results were obtained after correcting for many variables, including GWG above the IOM recommendation, as well as for pre-eclampsia and gestational diabetes mellitus in present pregnancy.

Table 2 shows categories of BMI and GWG in cases of hypertension or diabetes (GH, PE, GDM-1, and GDM-2). In all cases, there were more women with pre-pregnancy obesity and overweight, compared to controls. In hypertension cases (GH and PE), there were more women with GWG above the IOM recommendation. In diabetes cases (GDM-1 and GDM-2), there were more women with GWG below the recommendation.

Table 3 shows multivariate relationships between differ categories of BMI and GWG and the risk of hypertension or diabetes in pregnancy. Maternal obesity before pregnancy was the strongest risk factor for all the diseases (GH, PE, GDM-1, and GDM-2), especially for the severe forms. The results were obtained after correcting for many variables including excessive GWG. The risk of GH and PE was higher for GWG above the range, but the risk of GDM-1 and GDM-2 was higher for GWG below the range. The result for GDM-2 was close to the materiality limit.

Table S3 shows that the results presented in Table 3 were sustained (or were stronger) in subgroups: the subgroup of GWG in the range of the IOM recommendations (for BMI analyses) and the subgroup of the normal BMI (for GWG analyses).

The effect of maternal edema on the results was negligible (Table S4).

**Figure 1:**
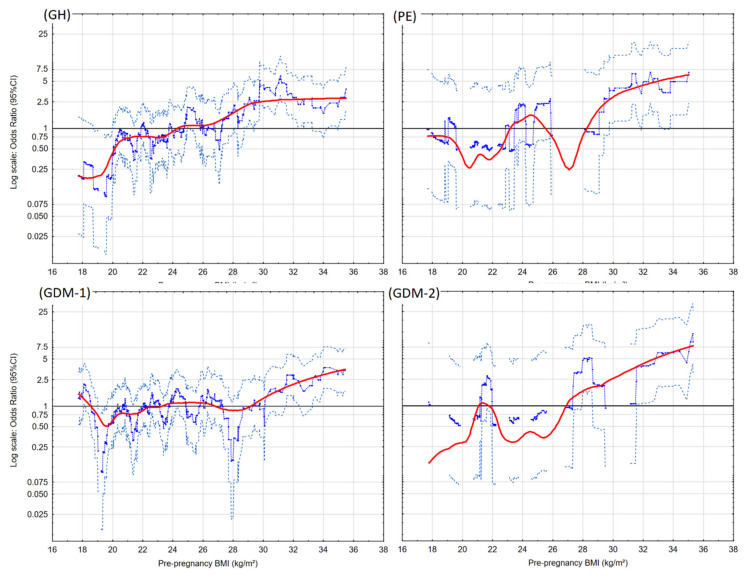
The risk profiles of hypertension in pregnancy (gestational hypertension (GH) and preeclampsia (PE)) and diabetes in pregnancy (gestational diabetes with dietary modification (GDM-1) and gestational diabetes with insulin therapy (GDM-2)), for pre-pregnancy body mass index (BMI). The graphs illustrate the changes in the crude risk (OR) of the diseases, estimated on a sliding window (for 30 observations) with respect to the BMI values. The blue points are the odds ratios of the diseases in the adopted window. The curves represent the risk profiles (the curves are smoothed with the Lowess method). The horizontal lines represent OR = 1.00. The light gray points represent the upper and lower limits of the 95% confidence intervals (CI).

Figure 1 presents the risk profiles of GH, PE, GDM-1, and GDM-2 for maternal BMI before pregnancy (kg/m^2^). The higher pre-pregnancy BMI was associated with the higher risk of studied diseases, especially of the severe forms (PE and GDM-2). The threshold point (of pre-pregnancy BMI) was 24.3 kg/m^2^, above which the risk of GH was higher, and 23.1 kg/m^2^, above which the risk of PE was higher (this first point). The threshold risk of development of GDM-2 occurred at lower BMI values (26.9 kg/m^2^), compared to GDM-1 (29.1 kg/m^2^).

## 4. Discussion

In our study, higher values of maternal BMI before pregnancy increased the risk of pregnancy complications such as hypertension and diabetes. Pre-pregnancy obesity was a strong (and statistically significant) risk factor of these diseases, to a greater extent than overweight or excessive gestational weight gain (GWG). Gestational weight gain (GWG) above the range (vs. GWG in the range) was a weak risk factor (and statistically insignificant) for studied diseases, and was associated with higher odds ratios of GH and PE, and lower odds ratios of GDM-1 and GDM-2.

Similar connections between obesity before pregnancy and hypertension or diabetes in pregnancy have been demonstrated in the literature [4,5,6,11,12,13,14,15,17,18,20,21,22,23]. Excessive gestational weight gain (GWG) was also found (in the literature) as a factor increasing preeclampsia/gestational hypertension risk, and the results were weaker than for pre-pregnancy obesity [6,22,23]. The relationships between excessive GWG, and a lower risk of gestational diabetes (GDM) were also found [16,17]. Cho et al. showed that an increase in GWG rate reduced the risk of GDM in late pregnancy, but not in early pregnancy [18]. The studies differed in research constructions, risks of the populations studied, sample sizes, definitions of BMI categories, and degree of adjustment by confounding variables.

Our study was based on the data from a prospective cohort study of women with a single pregnancy. The body mass index (BMI) was defined in line with the World Health Organization (WHO) guidelines, and the categories of weight gain in pregnancy (GWG) were defined in line with the 2009 IOM guidelines. The risk was corrected by many confounding variables, including GWG above the range (in the study of BMI) and pre-pregnancy BMI (in the study of GWG). The general profile of our cohort, especially for GH (12.4%) and GDM-1 (13.7%), was in accordance with results in the literature [4,7,10].

Several results we obtained need to be highlighted.

Firstly, the results for risk of severe forms of the pregnancy complications (PE and GDM-2) for pre-pregnancy obesity were stronger than for isolated gestational hypertension (GH) and diabetes with diet modification GDM-1. However, the number of PE and GDM-2 cases was small (24 (2.6%) and 21 (2.3%), respectively). Our methodology was probably influenced by the low incidence of PE in our study; we excluded some risk factors of preeclampsia, such as multiple pregnancy and pre-existing chronic diseases [7,24]. Other PE/GH risk factors such as infertility treatment, hypertension in previous pregnancy, and smoking were more common in the women who developed GH or PE than in normotensive women; however, the number of women with these risk factors was low. Many studies have shown that assisted reproductive techniques, including in vitro fertilization, are associated with a higher risk of hypertension in pregnancy, including various preeclampsia phenotypes [7,24,25,26]. The effect of smoking on the risk of preeclampsia is ambiguous, but, in our previous study, smoking in the first trimester increased the risk of GH and PE [27]. In the current study, these variables were among the factors correcting the results of GH and PE risk. In our cohort, the low incidence of insulin-treated diabetes (GDM-2) may be associated with the exclusion of pre-existing diabetes, the low number of women with diabetes in early pregnancy, and the low incidence of obesity. The incidence of obesity in our cohort (10.8%) is consistent with the data from Polish studies. The studies of overweight and obesity among adults in Poland during 2013–2014 show that the average prevalence of obesity was 9.2% among women 20–34 years old and 17.0% among women 35–44 [28]. A large retrospective analysis of hospital data from the UK shows that the average prevalence of obesity was 14.6% among women with a single pregnancy [29].

Secondly, our results attract attention because the pre-pregnancy BMI threshold points (above which an increase in the risk of the pregnancy complications was recorded) are within the upper limit of the normal BMI (18.5–24.99 kg/m^2^) and within the limit of overweight. The threshold point for the increase in the odds ratios of isolated gestational hypertension (GH) was 24.3 kg/m^2^, and for the increase in the preeclampsia (PE) odds ratios was 23.1 kg/m^2^ (this first point) (Figure 1). We found that the threshold risk of development of gestational diabetes requiring insulin (GDM-2) occurred at lower BMI values (26.9 kg/m^2^), compared to gestational diabetes without insulin (29.1 kg/m^2^). This requires examination in a larger cohort.

Thirdly, we found that gestational weight gain (GWG) above the range (vs. GWG in the range) was associated with higher odds ratios of hypertension forms (GH and PE), and lower odds ratios of diabetes forms (GDM-1 and GDM-2) (Table 3). The results were statistically insignificant. However, we also found that GWG below the range (vs. GWG in the range) was associated with over 2–3 times higher risk of GDM-1 (AOR = 2.53; *p* < 0.001) and GDM-2 (AOR = 3.43; *p* = 0.046). The results were statistically significant, but the result for GDM-2 was close to the materiality limit. This requires examination in a larger cohort.

Importantly, all the results were obtained after correcting for many confounders and in the subgroups (Table 3 and Table S3). The effect of maternal edema on the results was negligible (Table S4).

Fourthly, we found that pregnancy hypertension and diabetes were associated with adverse newborn outcomes, including a lower gestational age of delivery and/or incorrect birth weight (Table 1 and Table S1). Our study shows the importance and influence of excessive pre-pregnancy weight on the risk of pregnancy complications like diabetes and hypertension which can impact fetal outcomes. At the same time, we also found that excessive BMI (≥ 25 kg/m^2^) was associated with a higher risk of adverse newborn outcomes (Table S2).

The mechanisms linking excessive weight with adverse pregnancy outcomes and complications are complex and not fully clear. The etiologies of studied diseases are not fully explained and many biomarkers may be involved [30,31]. Because we did not evaluate any biomarkers in this analysis, we refer to the main pathogenetic mechanisms. Overweight and obesity impact insulin signaling pathways (and β-cell insulin production) [10]. Overweight and obesity have been associated with inflammation, oxidative stress, and insulin resistance, as well as with changes in the levels of various bioactive compounds, e.g., lipids, insulin, leptin, adipokines, and some cytokines [1,2,10]. These bioactive compounds participate in numerous metabolic pathways and adversely affect the endothelium [10,32]. Inflammation, oxidative stress, and endothelial dysfunction are important elements of pregnancy hypertension and diabetes [8,10]. The relationships between non-optimal levels of nutrients involved in oxidative balance and/or inflammatory processes and risk of hypertension or diabetes in pregnancy were also found. In our previous studies in the same cohort, we found the relationships between a higher risk of PIH and lower levels of first trimester selenium (Se), iron (Fe), and copper (Cu) in the serum [31,33,34]. In another study we conducted, we found the relationships between GDM and lower Fe and higher Cu levels [30]. We also found that excessive pre-pregnancy BMI was associated with lower Se and Fe levels and higher Cu levels [30,31].

The connections between higher gestational weight gain (GWG) and unfavorable pregnancy results are difficult to investigate. Firstly, it is known that the appropriate gestational weight gain differs depending on the pre-pregnancy BMI. It was also found that the relationship between GWG and a specific complication may vary between pregnancy periods [18]. It is not certain to what extent weight gain is the result of increased body fat, and to what degree it results from fluid retention. It is not explained to what extent the increase in GWG is a cause of higher risk of pregnancy-induced hypertension/preeclampsia, because possible edema associated with hypertension and/or protein loss also increases GWG. Secondly, some light on the inverse relationship between GWG and the risk of gestational diabetes may be shed by the studies conducted by Cho et al. who found that an increase in the rate of GWG in late pregnancy reduced the risk of gestational diabetes, but the increase in GWG in early pregnancy increased the risk of gestational diabetes [18]. It is possible that after the diagnosis of diabetes in 24–28 pregnancy week (based on the guidelines) weight gain is limited as a result of implementing typical recommendations for changing diet, physical activity, or treatment with insulin. Importantly, in our study, the effect of edema on the results was negligible (Table S3).

### Advantages and Limitations

Our research has several advantages. It was based on the data from a prospective cohort. We calculated the minimum sample size before recruitment. We excluded chronic diseases during recruitment (except for overweight and obesity), reducing the number of risk factors having negative influence on the results. We applied multivariate logistic regression and adjusted the risk for existing confounding variables. However, although we accumulated many data, other factors are possible. We also assessed the impact of maternal edema on the risks we examined. In the joint cohort, we evaluated the pre-pregnancy BMI and gestational weight gain simultaneously, and at the same time assessed two important pregnancy complications and its forms. We evaluated the whole cohort and the subgroups.

We are also aware of the limitations our research. The participants reported their body weight before pregnancy themselves, but this practice is often used. Both methods, examining the BMI or GWG by means of categorical variables or continuous variables have their limitations. Information about edema came from medical records. Sizes of subgroups were smaller, but the results of risk after adjustment were statistically significant. We did not assess the degree of glucose control, but the number of cases of diabetes treated with insulin was small. Despite the limitations of our study, we believe that the methodology we chose allowed systematically assessing and comparing two important risk factors of adverse pregnancy outcomes.

## 5. Conclusions

This study highlights the importance and influence of excessive pre-pregnancy maternal weight on the risk of pregnancy complications such as diabetes and hypertension, which can impact fetal outcomes.

Continued emphasis should be placed on the normalization of maternal weight before pregnancy, because higher pre-pregnancy BMI values (according to the threshold points covering the upper limit of normal BMI) were strongly associated with a higher risk of pregnancy-induced hypertension and gestational diabetes mellitus.

Our results for severe forms of hypertension and diabetes in pregnancy require examination in a larger cohort.

## Figures and Tables

**Table 1:** Characteristics of newborn outcomes in the mothers with hypertension or diabetes in pregnancy.

	Controls	Cases	
Characteristics	*n* (%)	*n* (%)	*p* *
	Normotensives (*n* = 775)	GH cases (*n* = 113)	
Birth Weight (g)	3416.5 (511.7); 3449.0	3174.1 (734.3); 3200.0	0.001
<10th Percentile	42 (5.4%)	21 (18.6%)	<0.001
10–90th Percentile	652 (84.1%)	75 (66.4%)	**
> 90th Percentile	81 (10.5%)	17 (15%)	0.038
Gestational Age (Week)	38.9 (1.6); 39.0	38.3 (2.2); 39.0	0.016
Birth < 37th Week	41 (5.3%)	11 (9.7%)	0.060
Birth < 34th Week	10 (1.3%)	7 (6.2%)	0.003
	Normotensives (*n* = 775)	PE cases (*n* = 24)	
Birth Weight (g)	3416.5 (511.7); 3449.0	2294.2 (927.5); 2445.0	<0.001
<10th Percentile	42 (5.4%)	9 (37.5%)	<0.001
10–90th Percentile	652 (84.1%)	14 (58.3%)	**
>90th Percentile	81 (10.5%)	1 (4.2%)	1.000
Gestational Age (Week)	38.9 (1.6); 39.0	35.1 (3.7); 36.0	<0.001
Birth < 37th Week	41 (5.3%)	13 (54.2%)	<0.001
Birth < 34th Week	10 (1.3%)	6 (25%)	<0.001
	Without diabetes (*n* = 766)	Diabetes GDM-1 (*n* = 125)	
Birth Weight (g)	3347.2 (588.3); 3400.0	3416.1 (547.8); 3400.0	0.470
<10th Percentile	59 (7.7%)	11(8.8%)	0.491
10–90th Percentile	633 (82.6%)	93 (74.4%)	**
>90th Percentile	74 (9.7%)	21 (16.8%)	0.014
Gestational Age (Week)	38.7 (2.0); 39.0	38.7 (1.5); 39.0	0.125
Birth < 37th Week	54 (7%)	9 (7.2%)	0.952
Birth < 34th Week	21 (2.7%)	1 (0.8%)	0.346
	Without diabetes (*n* = 766)	Diabetes GDM-2 (*n* = 21)	
Birth Weight (g)	3347.2 (588.3); 3400.0	3358.6 (837.8); 3300.0	0.771
<10th Percentile	59 (7.7%)	2 (9.5%)	0.651
10–90th Percentile	633 (82.6%)	15 (71.4%)	**
>90th Percentile	74 (9.7%)	4 (19%)	0.137
Gestational Age (Week)	38.7 (2.0); 39.0	38.0 (2.2); 38.0	0.037
Birth < 37th Week	54 (7%)	2 (9.5%)	0.656
Birth < 34th Week	21 (2.7%)	1 (4.8%)	0.453

* For binomial categories comparison, we used the Pearson chi-square test (or Fisher exact test when Cochran assumption was not met), and we used the Mann–Whitney U test (because normality assumption was not met) for comparisons of continuous variables (*p* < 0.05 was assumed to be significant). ** Reference category. GH, gestational hypertension; PE, preeclampsia; GDM-1, gestational diabetes mellitus with dietary modification; GDM-2, gestational diabetes with additional insulin therapy GDM-2; BMI, body mass index.

**Table 2:** Characteristics of pre-pregnancy BMI and gestational weight gain in the mothers with hypertension or diabetes in pregnancy.

	Controls	Cases	
Characteristics	*n* (%)	*n* (%)	*p* *
	Normotensives (*n* = 775)	GH cases (*n* = 113)	
Pre-pregnancy BMI (kg/m^2^)	23.3 (4.1); 22.5	26.7 (5.3); 25.5	<0.001
Obesity	58 (7.5%)	31 (27.4%)	<0.001
Overweight	139 (17.9%)	30 (26.5%)	
Normal Pre-Pregnancy BMI	534 (68.9%)	51 (45.1%)	
Underweight	44 (5.7%)	1 (0.9%)	
GWG (kg)	13.4 (5.3); 13.0	14.6 (8); 14,0	0.115
GWG Above Recommendation	263 (33.9%)	62 (54.9%)	<0.001
GWG in Range of Recommendation **	301 (38.8%)	29 (25.7%)	
GWG Below Recommendation	211 (27.2%)	22 (19.5%)	
	Normotensives (*n* = 775)	PE cases (*n* = 24)	
Pre-Pregnancy BMI (kg/m^2^)	23.3 (4.1); 22.5	26.5 (6.2); 25.0	0.008
Obesity	58 (7.5%)	9 (37.5%)	<0.001
Overweight	139 (17.9%)	4 (16.7%)	
Normal Pre-Pregnancy BMI	534 (68.9%)	9 (37.5%)	
Underweight	44 (5.7%)	2 (8.3%)	
GWG (kg)	13.4 (5.3); 13.0	15.1(8.2); 14.3	0.612
GWG Above Recommendation	263 (33.9%)	11 (45.8%)	0.258
GWG in Range of Recommendation **	301 (38.8%)	8 (33.3%)	
GWG Below Recommendation	211 (27.2%)	5 (20.8%)	
	Without diabetes (*n* = 766)	Diabetes GDM-1 (*n* = 125)	
Pre-Pregnancy BMI (kg/m^2^)	23.5 (4.2); 22.6	25.0 (5.2); 23.9	0.004
Obesity	66 (8.6%)	25 (20.0%)	0.007
Overweight	147 (19.2%)	21 (16.8%)	
Normal Pre-Pregnancy BMI	515 (67.2%)	71 (56.8%)	
Underweight	38 (5.0%)	8 (6.4%)	
GWG (kg)	14.1 (5.5); 14.0	11.2 (6.7); 11.0	<0.001
GWG Above Recommendation	298 (38.9%)	33 (26.4%)	<0.001
GWG in Range of Recommendation **	292 (38.1%)	39 (31.2%)	
GWG Below Recommendation	176 (23.0%)	53 (42.4%)	
	Without diabetes (*n* = 766)	Diabetes GDM-2 (*n* = 21)	
Pre-Pregnancy BMI (kg/m^2^)	23.5 (4.2); 22.6	27.9 (7.0); 28.0	0.003
Obesity	66 (8.6%)	7 (33.3%)	0.001
Overweight	147 (19.2%)	5 (23.8%)	
Normal Pre-Pregnancy BMI	515 (67.2%)	8 (38.1%)	
Underweight	38 (5.0%)	1 (4.8%)	
GWG (kg)	14.1 (5.5); 14.0	9.0 (5.0); 9.0	<0.001
GWG Above Recommendation	298 (38.9%)	5 (23.8%)	0.041
GWG in Range of Recommendation **	292 (38.1%)	7 (33.3%)	
GWG Below Recommendation	176 (23.0%)	9 (42.9%)	

* The Cochran–Armitage test was used for categorical ordered categories for trend comparisons (*p* <0.05 was assumed to be significant), and we used the Mann–Whitney U test (because normality assumption was not met) for comparisons of continuous variables ** GWG, gestational weight gain, defined in accordance with the recommendations of Institute of Medicine (IOM) from 2009; GH, gestational hypertension; PE, preeclampsia; GDM-1, gestational diabetes mellitus with dietary modification; GDM-2, gestational diabetes with additional insulin therapy GDM-2; BMI, body mass index.

**Table 3:** The adjusted odds ratios of hypertension and diabetes in pregnancy for various categories of maternal weight.

	The Odds Ratios of the Diseases for Weight Categories			
	Cases/ Controls	OR (95% CI:); *p*	AOR-c * (95% CI:); *p*	
Gestational Hypertension (GH) **				
Obesity	31/58	5.60 (3.32–9.43); < 0.001	4.94 (2.77–8.81); < 0.001	
Overweight	30/139	2.26 (1.39–3.68); 0.001	2.09 (1.21–3.60); 0.008	
Underweight	1/44	0.24 (0.03–1.76); 0.16	0.29 (0.04–2.17); 0.226	
Normal Pre-Pregnancy BMI	51/534	1	1	
GWG Above the Range	62/263	2.45 (1.53–3.92); < 0.001	1.71 (1.01–2.89); 0.045	
GWG in the Range	29/301	1	1	
GWG Below the Range	22/211	1.08 (0.61–1.94); 0.790	1.02 (0.54–1.91); 0.950	
Preeclampsia (PE) **				
Obesity	9/58	9.21 (3.52–24.11); < 0.001	8.61 (3.05–24.36); < 0.001	
Overweight	4/139	1.71 (0.52–5.63); 0.379	1.91 (0.53–6.87); 0.324	
Underweight	2/44	2.70 (0.57–12.87); 0.213	2.95 (0.57–15.16); 0.195	
Normal Pre-Pregnancy BMI	9/534	1	1	
GWG Above the Range	11/263	1.57 (0.62–3.97); 0.337	1.14 (0.41–3.12); 0.803	
GWG in the Range	8/301	1	1	
GWG Below the Range	5/211	0.89 (0.29–2.76); 0.842	0.80 (0.24–2.65); 0.716	
Gestational Diabetes GDM-1 ***				
Obesity	25/66	2.75 (1.63–4.64); < 0.001	2.99 (1.71–5.25); < 0.001	
Overweight	21/147	1.04 (0.62–1.74); 0.893	1.24 (0.71–2.14); 0.452	
Underweight	8/38	1.53 (0.69–3.41); 0.301	1.53 (0.66–3.52); 0.322	
Normal Pre-Pregnancy BMI	71/515	1	1	
GWG Above the Range	33/298	0.83 (0.51–1.36); 0.454	0.74 (0.44–1.24); 0.245	
GWG in the Range	39/292	1	1	
GWG Below the Range	53/176	2.26 (1.43–3.55); < 0.001	2.53 (1.58–4.06); < 0.001	
Gestational Diabetes GDM-2 ***				
Obesity	7/66	6.83 (2.4–19.44); < 0.001	11.88 (3.67–38.48); < 0.001	
Overweight	5/147	2.19 (0.71–6.79); 0.175	2.29 (0.58–9.06); 0.238	
Underweight	1/38	1.69 (0.21–13.9); 0.624	2.66 (0.3–23.74); 0.381	
Normal Pre-Pregnancy BMI	8/515	1	1	
GWG Above the Range	5/298	0.70 (0.22–2.23); 0.546	0.76 (0.21–2.77); 0.672	
GWG in the Range	7/292	1	1	
GWG Below the Range	9/176	2.13 (0.78–5.83); 0.14	3.43 (1.02–11.5); 0.046	

* AOR (adjusted odds ratios) (CI, confidence intervals) were calculated in the multivariate logistic regression, and the *p*-value was calculated using the Wald test (*p* <0.05 was assumed to be significant). ** AOR-c: The odds ratios were adjusted for maternal age, being primiparous, GWG above the range of the recommendation (in the analyses of BMI) or pre-pregnancy BMI (in the analyses of GWG), smoking, hypertension in previous pregnancies, and infertility treatment. *** AOR-c: The odds ratios were adjusted for maternal age, being primiparous, GWG above the range of the recommendation (in the analyses of BMI) or pre-pregnancy BMI (in the analyses of GWG), and gestational diabetes in previous pregnancies; GDM-1, gestational diabetes with dietary modification; GDM-2, gestational diabetes with insulin therapy; BMI, body mass index; GWG, gestational weight gain defined in accordance with the recommendations of Institute of Medicine (IOM) from 2009.

## Supplementary Materials

The following are available online at https://www.mdpi.com/2077-0383/9/6/1980/s1, Table S1: General characteristics in the mothers with hypertension or diabetes in pregnancy; Table S2: Multivariate relationships between excessive pre-pregnancy BMI and adverse newborn outcomes; Table S3: The adjusted odds ratios of GH, PE, GDM-1, and GDM-2 for categories of maternal weight, in the subgroups; Table S4: The adjusted odds ratios of GH, PE, GDM-1, and GDM-2 for categories of maternal weight, in model with maternal edema.
