# Supplementary material for: Pre-Pregnancy Obesity, Excessive Gestational Weight Gain, and the Risk of Pregnancy-Induced Hypertension and Gestational Diabetes Mellitus
Source: J Clin Med. 2020 Jun 24;9(6):1980. doi: 10.3390/jcm9061980 (PMC7355601; doi:10.3390/jcm9061980)
Supplement: Supplementary file 1 [file jcm-09-01980-s001.pdf]

Supplementary Materials

Table S1. General characteristics in the mothers with hypertension or diabetes in pregnancy.

	Controls	Cases	
**Maternal characteristics**	**Mean (SD); Median/ *n* (%)**	**Mean (SD); Median/ *n* (%)**	***p* ***
	Normotensives (*n* = 775)	GH cases (*n* = 113)	
Maternal age (years)	33.5 (4.8); 35.0	35.0 (4.3); 36.0	0.005
Primiparous	318 (41.0%)	53 (46.9%)	0.237
Hypertension in previous pregnancy	4 (0.5%)	12 (10.6%)	<0.00001
Infertility treatment	29 (3.7%)	8 (7.1%)	0.097
Smokers in the first trimester	37 (4.8%)	17 (15.0%)	0.00002
Pre-pregnancy BMI (kg/m²)	23.3 (4.1); 22.5	26.7 (5.3); 25.5	< 0.001
GWG (kg)	13.4 (5.3); 13.0	14.6 (8); 14,0	0.115
Gestational age at delivery (week)	38.9 (1.6); 39.0	38.3 (2.2); 39.0	0.016
Newborn birthweight (g)	3416.5 (511.7); 3449.0	3174.1 (734.3); 3200.0	0.001
Gestational diabetes mellitus	121 (15.6%)	22 (19.5%)	0.298
	Normotensives (*n* = 775)	PE cases (*n* = 24)	
Maternal age (years)	33.5 (4.8); 35.0	34.1 (5.0); 35.0	0.434
Primiparous	318 (41.0%)	12 (50.0%)	0.380
Hypertension in previous pregnancy	4 (0.5%)	3 (12.5%)	<0.00001
Infertility treatment	29 (3.7%)	3 (12.5%)	0.031
Smokers in the first trimester	37 (4.8%)	3 (12.5%)	0.084
Pre-pregnancy BMI (kg/m²)	23.3 (4.1); 22.5	26.5 (6.2); 25.0	0.008
GWG (kg)	13.4 (5.3); 13.0	15.1(8.2); 14.3	0.612
Gestational age at delivery (week)	38.9 (1.6); 39.0	35.1 (3.7); 36.0	< 0.001
Newborn birthweight (g)	3416.5 (511.7); 3449.0	2294.2 (927.5); 2445.0	< 0.001
Gestational diabetes mellitus	121 (15.6%)	3 (12.5%)	0.678
PE beginning ≥34th week	-	13 (54.2%)	-
	Without diabetes (*n* = 766)	Diabetes GDM-1 (*n* = 125)	
Maternal age (years)	33.5 (4.8); 34.0	35.1 (4.3); 36.0	<0.001
Primiparous	318 (41.5%)	59 (47.2%)	0.233
Diabetes in previous pregnancy	3 (0.4%)	3 (2.4%)	0.039
Pre-pregnancy BMI (kg/m²)	23.5 (4.2); 22.6	25.0 (5.2); 23.9	0.004
GWG (kg)	14.1 (5.5); 14.0	11.2 (6.7); 11.0	<0.001
Gestational age at delivery (week)	38.7 (2.0); 39.0	38.7(1.5); 39.0	0.125
Newborn birthweight (g)	3347.2 (588.3); 3400.0	3416.1 (547.8); 3400.0	0.470
Pregnancy-induced hypertension	112 (14.6%)	20 (16.0%)	0.687
	Without diabetes (*n* = 766)	Diabetes GDM-2 (*n* = 21)	
Maternal age (years)	33.5 (4.8); 34.0	34.9 (4.4); 36.0	0.176
Primiparous	318 (41.5%)	5 (23.8%)	0.104
Diabetes in previous pregnancy	3 (0.4%)	5 (23.8%)	<0.001
Prepregnancy BMI (kg/m²)	23.5 (4.2); 22.6	27.9 (7.0); 28.0	0.003
GWG (kg)	14.1 (5.5); 14.0	9.0 (5.0); 9.0	<0.001
Gestational age at delivery (week)	38.7 (2.0); 39.0	38.0 (2.2); 38.0	0.037
Newborn birthweight (g)	3347.2 (588.3); 3400.0	3358.6 (837.8); 3300.0	0.771
Pregnancy-induced hypertension	112 (14.6%)	5 (23.8%)	0.222

* The Mann–Whitney U test was used for comparisons of continuous variables, and the Pearson chi-square test (or Fisher exact test when Cochran assumption was not met) was used for binomial categories ($p<0.05$ was assumed to be significant); GH, gestational hypertension; PE, preeclampsia; GDM-1, gestational diabetes with dietary modification; GDM-2, gestational diabetes with insulin therapy; BMI, body mass index; GWG, gestational weight gain.

Table S2. Multivariate relationships between excessive pre-pregnancy BMI and adverse newborn outcomes.

The Odds Ratios of Incorrect Birth Weight for Excessive BMI			
	**Cases/ Controls**	**OR (95% CI:); *p***	**AOR-c * (95% CI:); *p***
Birth weight >90th percentile **			
Pre-pregnancy BMI ≥25 kg/m^2	47/201	2.46 (1.59–3.79); <0.001	1.69 (1.05–2.73); 0.031
Normal BMI (18.5–24.99 kg/m^2)	48/504	1	1
Birth weight >4000 g ***			
Pre-pregnancy BMI ≥25 kg/m^2	49/197	2.93 (1.89–4.54); <0.001	1.83 (1.12–3.01); 0.016
Normal BMI (18.5–24.99 kg/m^2)	44/518	1	1
Birth weight <10th percentile **			
Pre-pregnancy BMI ≥25 kg/m^2	23/201	1.37 (0.81–2.34); 0.245	1.17 (0.64–2.13); 0.62
Normal BMI (18.5–24.99 kg/m^2)	42/504	1	1
Birth weight <2500 g ***			
Pre-pregnancy BMI ≥25 kg/m^2	25/197	2.05 (1.19–3.55); 0.01	1.60 (0.67–3.86); 0.293
Normal BMI (18.5–24.99 kg/m^2)	32/518	1	1
Birth <37th week ** **			
Pre-pregnancy BMI ≥25 kg/m^2	24/247	1.46 (0.86–2.5); 0.164	1.62 (0.87–3); 0.126
Normal BMI (18.5–24.99 kg/m^2)	37/557	1	1
Birth <34th week ** **			
Pre-pregnancy BMI ≥25 kg/m^2	11/260	2.47 (1.04–5.89); 0.041	3.22 (1.14–9.12); 0.028
Normal BMI (18.5–24.99 kg/m^2)	10/584	1	1

* AOR (adjusted odds ratios) (CI, confidence intervals) were calculated in the multivariate logistic regression, and the p-value was calculated using the Wald test ($p<0.05$ was assumed to be significant). ** AOR-c: The analysis covered cases and newborns within 10th–90th percentile, and the odds ratios were adjusted for maternal age, primiparous, GWG above the range of the IOM recommendation, smoking in the first trimester, maternal height, fetal sex, pre-eclampsia in present pregnancy, and diabetes mellitus in present pregnancy. *** AOR-c: The analysis covered cases and newborns within 2500–4000 g, and the odds ratios were adjusted for maternal age, primiparous, GWG above the range of the IOM recommendation, smoking in the first trimester, maternal height, fetal sex, pre-eclampsia in present pregnancy, diabetes mellitus in present pregnancy, and gestational age at delivery. **** AOR-c: The analysis covered cases and births ≥37th week, and the odds ratios were adjusted for maternal age, primiparous, GWG above the range of the IOM recommendation, smoking in the first trimester, fetal sex, pre-eclampsia in present pregnancy, cesarean section, and premature rupture of membranes;. BMI, body mass index; GWG, gestational weight gain defined in accordance with the recommendations of Institute of Medicine (IOM) from 2009.

Table S3. The adjusted odds ratios of GH, PE, GDM-1, and GDM-2 for categories of maternal weight, in the subgroups.

			The Odds Ratios of Hypertension and Diabetes in Pregnancy	
	**Cases/ Controls**	**In the Whole Cohort OR (95% CI:); *p***	**In the Whole Cohort AOR-c (95% CI:); *p* ****	**In the Subgroups * AOR-c (95% CI:); *p*****
Gestational hypertension (GH) ***				
OBESITY	31/58	5.60 (3.32–9.43); <0.001	4.94 (2.77–8.81); <0.001	8.21 (2.47–27.26); 0.001
Overweight	30/139	2.26 (1.39–3.68); 0.001	2.09 (1.21–3.60); 0.008	5.81 (2.11–15.99); 0.001
Underweight	1/44	0.24 (0.03–1.76); 0.16	0.29 (0.04–2.17); 0.226	NA
Normal pre-pregnancy BMI	51/534	1	1	1
GWG above the range	62/263	2.45 (1.53–3.92); <0.001	1.71 (1.01–2.89); 0.045	3.00 (1.39–6.49); 0.005
GWG in the range	29/301	1	1	1
Preeclampsia (PE) ***				
OBESITY	9/58	9.21 (3.52–24.11); <0.001	8.61(3.1–24.36); <0.001	21.06 (2.82–157.23); 0.003
Overweight	4/139	1.71 (0.52–5.63); 0.379	1.91 (0.53–6.87); 0.324	3.65 (0.3–44.29); 0.310
Underweight	2/44	2.70 (0.57–12.87); 0.213	2.95(0.57–15.16); 0.195	11.27(1.18–107.36); 0.035
Normal pre-pregnancy BMI	9/534	1	1	1
GWG above the range	11/263	1.57 (0.62–3.97); 0.337	1.14 (0.41–3.12); 0.803	3.15 (0.52–19.1); 0.211
GWG in the range	8/301	1	1	1
Diabetes GDM-1 ** **				
OBESITY	25/66	2.75 (1.63–4.64); <0.001	2.99(1.71–5.25); <0.001	2.69 (0.87–8.25); 0.085
Overweight	21/147	1.04 (0.62–1.74); 0.893	1.24 (0.71–2.14); 0.452	1.93 (0.79–4.70); 0.150
Underweight	8/38	1.53 (0.69–3.41); 0.301	1.53 (0.66–3.52); 0.322	1.52 (0.39–5.92); 0.544
Normal pre-pregnancy BMI	71/515	1	1	1
GWG above the range	33/298	0.83 (0.51–1.36); 0.454	0.74 (0.44–1.24); 0.245	0.69 (0.33–1.47); 0.339
GWG in the range	39/292	1	1	1
Diabetes GDM-2 ** **				
OBESITY	7/66	6.83 (2.4–19.44); <0.001	11.88(3.7–38.5); <0.001	35.84 (4.2–303.1); 0.001
Overweight	5/147	2.19 (0.71–6.79); 0.175	2.29 (0.58–9.06); 0.238	4.51 (0.46–44.68); 0.198
Underweight	1/38	1.69 (0.21–13.9); 0.624	2.66 (0.3–23.74); 0.381	NA
Normal pre-pregnancy BMI	8/515	1	1	1
GWG above the range	5/298	0.70 (0.22–2.23); 0.546	0.76 (0.21–2.77); 0.672	1.13 (0.07–19.08); 0.930
GWG in the range	7/292	1	1	1

* The analyses of pre-pregnancy BMI were conducted in the subgroup of GWG in the range of the IOM recommendations, and the analyses of GWG were carried out in the subgroup of pre-pregnancy

BMI in the norm. ** adjusted odds ratios (CI, confidence intervals) were calculated in the multivariate logistic regression, and the p-value was calculated using the Wald test (p <0.05 was assumed to be significant). *** AOR-c: The odds ratios were adjusted for maternal age, primiparous, GWG above the range of the recommendation (in the analyses of BMI) or pre-pregnancy BMI (in the analyses of GWG), smoking, hypertension in previous pregnancy, and infertility treatment. **** AOR-c: The odds ratios were adjusted for maternal age, primiparous, GWG above the range of the recommendation (in the analyses of BMI) or pre-pregnancy BMI (in the analyses of GWG), and diabetes in previous pregnancy. GDM-1, gestational diabetes with dietary modification; GDM-2, gestational diabetes with insulin therapy; BMI, body mass index; GWG, gestational weight gain defined in accordance with the recommendations of Institute of Medicine (IOM) from 2009. NA: data not available (no case).

Table S4. The adjusted odds ratios of GH, PE, GDM-1, and GDM-2 for categories of maternal weight, in model with maternal edema.

			**The Odds Ratios of Hypertension and Diabetes in Pregnancy**	
	**Cases/ Controls**	**OR (95% CI:); *p***	**AOR-a* (95% CI:); *p***	**AOR-b** (95% CI:); *p***
Gestational hypertension (GH)				
Obesity	31/58	5.60 (3.32–9.43); <0.001	4.41 (2.55–7.61); <0.001	4.45 (2.57–7.70); <0.001
Overweight	30/139	2.26 (1.39–3.68); 0.001	1.82 (1.09–3.05); 0.022	1.88 (1.12–3.15); 0.017
Underweight	1/44	0.24 (0.03–1.76); 0.16	0.27 (0.04–2.03); 0.204	0.25 (0.03–1.91); 0.182
Normal pre-pregnancy BMI	51/534	1	1	1
GWG above the range	62/263	2.45 (1.53–3.92); <0.001	2.00 (1.22–3.29); 0.006	1.86 (1.13–3.08); 0.015
GWG in the range	29/301	1	1	1
Preeclampsia (PE)				
OBESITY	9/58	9.21 (3.52–24.11); <0.001	8.38 (3.1–22.66); <0.001	7.53 (2.69–21.05); <0.001
Overweight	4/139	1.71 (0.52–5.63); 0.379	1.53 (0.44–5.30); 0.499	1.67 (0.48–5.83); 0.425
Underweight	2/44	2.70 (0.57–12.87); 0.213	2.81 (0.57–13.79); 0.204	2.33 (0.45–12.04); 0.312
Normal pre-pregnancy BMI	9/534	1	1	1
GWG above the range	11/263	1.57 (0.62–3.97); 0.337	1.31 (0.51–3.40); 0.575	0.92 (0.34–2.52); 0.872
GWG in the range	8/301	1	1	1
Gestational diabetes GDM-1				
OBESITY	25/66	2.75 (1.63–4.64); <0.001	3.05 (1.74–5.33); <0.001	3.04 (1.74–5.33); <0.001
Overweight	21/147	1.04 (0.62–1.74); 0.893	1.25 (0.72–2.16); 0.426	1.25 (0.72–2.16); 0.423
Underweight	8/38	1.53 (0.69–3.41); 0.301	1.61 (0.70–3.66); 0.26	1.61 (0.70–3.66); 0.260
Normal pre-pregnancy BMI	71/515	1	1	1
GWG above the range	33/298	0.83 (0.51–1.36); 0.454	0.70 (0.42–1.17); 0.176	0.68 (0.40–1.15); 0.147
GWG in the range	39/292	1	1	1

Gestational diabetes GDM-2				
OBESITY	7/66	6.83 (2.4–19.44); <0.001	8.48(2.87–25.01); <0.001	8.19 (2.76–24.26); <0.001
Overweight	5/147	2.19 (0.71–6.79); 0.175	3.04 (0.93–9.91); 0.065	3.07 (0.94–10.00); 0.063
Underweight	1/38	1.69 (0.21–13.9); 0.624	1.87 (0.22–15.82); 0.567	1.85 (0.22–15.74); 0.572
Normal pre-pregnancy BMI	8/515	1	1	1
GWG above the range	5/298	0.70 (0.22–2.23); 0.546	0.53 (0.16–1.73); 0.290	0.55 (0.17–1.81); 0.325
GWG in the range	7/292	1	1	1

AOR (adjusted odds ratios) (CI, confidence intervals) were calculated in the multivariate logistic regression, and the p-value was calculated using the Wald test ($p<0.05$ was assumed to be significant). * AOR-a: The odds ratios were adjusted for maternal age, primiparous, and GWG above the range of the recommendation (in the analyses of BMI) or pre-pregnancy BMI (in the analyses of GWG). ** AOR-b: The odds ratios were adjusted for maternal edema, maternal age, primiparous, and GWG above the range of the recommendation (in the analyses of BMI) or pre-pregnancy BMI (in the analyses of GWG). GDM-1, gestational diabetes with dietary modification; GDM-2, gestational diabetes with insulin therapy; BMI, body mass index; GWG, gestational weight gain defined in accordance with the recommendations of Institute of Medicine (IOM) from 2009.
